# Antecedents, outcomes and measurement of work related-cognition in non-work time: A multistudy report using the work-related rumination questionnaire in two languages

**DOI:** 10.3389/fpsyg.2023.1013744

**Published:** 2023-03-02

**Authors:** Roman Pauli, Petra Maria Gaum, Mark Cropley, Jessica Lang

**Affiliations:** ^1^Faculty of Medicine, Institute for Occupational, Social and Environmental Medicine, RWTH Aachen University, Aachen, Germany; ^2^Faculty of Health and Medical Sciences, School of Psychology, University of Surrey, Guildford, England, United Kingdom

**Keywords:** affective rumination, problem-solving pondering, detachment, job stress, measurement invariance

## Abstract

According to the perseverative cognition hypothesis, prolonged activation for example, *via* work-related rumination impairs recovery and thereby poses a risk to employee health. The extent to which gender, age, occupation or longitudinal stress exposure may alter work-related rumination is an ongoing debate. Whether group or longitudinal comparisons of work-related rumination are valid, however, has never been tested. In this multistudy report, we therefore investigated measurement invariance of the widely used Work-Related Rumination Questionnaire (WRRQ) across gender, age, occupation, and longitudinal measurements by performing secondary analyses of preexisting data on work-related rumination. We examined the psychometric properties of WRRQ measurements in two languages and expand knowledge about the nomological network of affective rumination, problem-solving pondering and detachment in relation to individual employee characteristics (e.g., personality, work engagement, commitment), job stressors (e.g., work intensity, decision latitude, social relations with colleagues and supervisors) and employee health outcomes (e.g., wellbeing, irritation, somatic symptoms). Multigroup confirmatory factor analyses showed partial scalar invariance of English and German WRRQ measurements and full scalar invariance across gender, age, occupation and over the period of 1 week (Study 1, *n* = 2,207). Correlation analyses supported criterion, convergent and discriminant validity of WRRQ measurements (Study 2, *n* = 4,002). These findings represent a prerequisite for comparisons of work-related cognition across groups and further the understanding of the antecedents and outcomes of different types of work-related cognition.

## Introduction

How individuals switch off from work in their leisure time and what prevents them from doing so are two questions at the heart of recovery research. Answering the first question, particularly psychological detachment acts to reduce fatigue, burnout or depression (Sonnentag et al., 2010; Sonnentag and Fritz, 2015). Therefore, it has been argued that successful recovery to a large extent depends on whether individuals are able to refrain from work-related thoughts (Cropley and Zijlstra, 2011). To this end, previous research addressed the second question of what keeps individuals from switching off by looking at work-related cognition. One key assumption is that perseverative cognition can prolong adverse effects of a stressor by sustaining that stressor’s cognitive representation (Brosschot et al., 2006). Given the prevalence of cognitive demands in contemporary workplaces, work-related thoughts have been characterized as one of the main drivers of inadequate recovery (Cropley and Zijlstra, 2011). Work-related cognition after work extends work-related mental efforts into leisure time, it thereby impairs recovery, and ultimately increases individuals’ vulnerability toward stress (Pravettoni et al., 2007; Meurs and Perrewé, 2011), as ruminative thinking is related to personality (Wang et al., 2013), physiological (Rydstedt et al., 2009; Vahle-Hinz et al., 2014; Zoccola et al., 2014), behavioral (Cropley et al., 2006, 2012; Berset et al., 2011; Vahle-Hinz et al., 2014; Syrek et al., 2017) and somatic outcomes (Verkuil et al., 2010, 2012; Ottaviani et al., 2016). It has therefore been argued that solely relying on mentally detaching from work ignores the relevance of the specific content and valence of thoughts which ultimately affect how individuals recover from work-related mental effort after work (e.g., Weigelt et al., 2019a; Jimenez et al., 2022) and to take into account work-related cognition as an important aspect of the recovery process.

The Work-Related Rumination Questionnaire (WRRQ; Cropley et al., 2012) is a widely used measure to assess work-related cognition *via* affective rumination, problem-solving pondering and (lack of) detachment. It has been translated into different languages (Firoozabadi et al., 2018; Sulak-Akyüz and Sulak, 2019; Rosario-Hernández et al., 2021; Pauli and Lang, 2021b; Lin and Bai, 2022) and utilized to compare measurements across different groups of employees (e.g., Pravettoni et al., 2007; Pauli and Lang, 2021a) or over time (Hamesch et al., 2014; Syrek et al., 2017; Kinnunen et al., 2019). However, it has yet to be established whether different measurement outcomes are attributable to actual differences between groups instead of differences in the measurement attributes (Steinmetz, 2013). In addition, an evaluation of the psychometric properties of the WRRQ is still pending. To this end, the present study extends prior research by investigating the psychometric properties, measurement invariance and nomological network of the WRRQ. In Study 1, we investigate the factor structure of work-related rumination and test for measurement invariance across language, gender, age and occupation groups as well as longitudinal measurements. In study 2, we investigate how antecedents (i.e., employee characteristics, job stressors) and outcomes (i.e., employee health) differentially relate to different types of work-related cognition. Implications for criterion, convergent and discriminant validity are discussed.

The results of both studies have important methodological implications for measuring rumination, as measurement invariance is a prerequisite for meaningful comparisons across different groups or over time (Kline, 2016). Due to the general trend of increasing job demands (Lohmann-Haislah, 2012) it is also important to know the differential relations of job stressors with rumination. In this regard, our findings have important theoretical implications as they provide additional knowledge about the nomological network, i.e., differential antecedents and outcomes, of affective rumination, problem-solving pondering and detachment.

## Conceptualization and measurement of work-related rumination

Rumination research draws upon Martin and Tesser’s (1996, p. 7) general definition of rumination as “a class of conscious thoughts that revolve around a common instrumental theme and that recur in the absence of immediate environmental demands requiring the thoughts.” Cropley and Zijlstra (2011) adapted this idea to the work context and in their conceptualization defined rumination as “thoughts directed to issues relating to work, that is/are repetitive in nature” (Cropley and Zijlstra, 2011, p. 6). In this regard, work-related rumination is reflected in a*ffective rumination*, characterized by thoughts about work that are negative in affect, intrusive, pervasive and recurrent (Cropley et al., 2012), *problem-solving pondering* encompassing unemotional thoughts revolving around a particular problem or previous work in search of a solution or improvement (Cropley and Zijlstra, 2011) and *detachment* as a state of absence of ruminative thoughts, that can be used to assess employees ability to switch-off from work (Cropley and Zijlstra, 2011). Therefore, we expect the following factor structure for an assessment of work-related cognition using the WRRQ

*H1*: A model of work-related rumination with the three factors (affective rumination, problem-solving pondering and detachment) best fits the empirical data.

Some groups are more susceptible to rumination than others and therefore differ in their exposure to the adverse effects of stressors. As work-related rumination prolongs job-related stress exposure, understanding differences in rumination across groups is crucial in preventing long term health impairments. In order to do so, previous research compared rumination across different groups of study participants: Women report more rumination and cognitive activation (Nolen-Hoeksema and Harrell, 2002) and show prolonged duration of cortisol activation after rumination (Shull et al., 2016) compared to men. The need for recovery increases with age (Kiss and de Meester, 2005) and older people report less ruminative thinking compared to other age groups (Sütterlin et al., 2012). The extent and nature of work-related thoughts varies across occupations, e.g., teaching professions having particular difficulties to mentally disengage from work (Aronsson et al., 2003; Cropley and Millward Purvis, 2003; Cropley et al., 2006) and creative workers reporting higher levels and different types of rumination compared to workers performing repetitive tasks (Pravettoni et al., 2007; Pauli and Lang, 2021a). However, differential measurement outcomes across groups do not *per se* indicate actual differences between the groups, but may also be due to differences in the measurements themselves (Steinmetz, 2013). Therefore, measurement invariance is a precondition for group comparisons, assuming that the measurements will function similarly irrespective of differences in the characteristics of the measurement objects (Kline, 2016). With regard to the comparison of rumination, e.g., across different occupations, it must consequently be ensured that differences in the scores measured in both groups are due to actual differences in rumination between occupations and not, as an example, due to the fact that unskilled workers interpret items differently than trained professionals. In addition to group differences, work-related rumination has been studied in relation to longitudinal outcomes (e.g., Hamesch et al., 2014; Syrek et al., 2017; Kinnunen et al., 2019) or to compare trait and state rumination (Shull et al., 2016). However, the stability of work-related rumination in individuals is still an open research question (Querstret and Cropley, 2012) as well as the stability of measurements of work-related rumination over time. These findings imply the hypothesis, that

*H2*: WRRQ measurements are invariant across language, gender, age, and occupational groups as well as longitudinally.

## Differential antecedents and outcomes of rumination

While in clinical health psychology, rumination encompasses repetitive thinking about emotional aspects of individual problems and failure, which inhibit individuals’ ability to engage in problem-solving and is related to depression (Lyubomirsky et al., 1998; Mellings and Alden, 2000; Nolen-Hoeksema et al., 2008; Zawadzki et al., 2013), others highlighted positive connotations of rumination, as positive work reflection and engaging in conversations about positive job events in nonwork time led to increased positive affect (Hicks and Diamond, 2008; Ilies et al., 2011; Sonnentag and Grant, 2012; Sonnentag et al., 2021). Rumination may thus provide a constructive strategy of coping with stressors, by anticipating their outcomes, as well as planning and rehearsing potential problem solutions (Watkins, 2008). Conceptually, affective rumination, problem-solving pondering and detachment—descending in that order—are contaminated with valence and content of off-job work-related thoughts (Jimenez et al., 2022). Weigelt et al. (2019a) pointed out that other than affective rumination, problem-solving pondering does not encompasses the negative affective quality of prolonged thinking, whereas detachment remains unspecific about the valence of work-related thought. In the following paragraphs, we elaborate on how these varying degrees of contamination cause different facets of the WRRQ to correlate differently with antecedents and outcomes of work-related thoughts.

### Antecedents

To start with the antecedents of work-related rumination, the following section outlines how the facets of the WRRQ relate to neuroticism, positive and negative affect, mindfulness, work engagement, commitment, recovery experiences as well as job stressors. The tendency to worry and react with negative emotionality is encapsulated in neuroticism (Barnhofer and Chittka, 2010), a personality trait that is described as an individual’s emotional stability and is responsible for affect regulation and individual adjustment to stress (Costa and McCrae, 1992). Neurotic symptoms are more prevalent in individuals high in rumination (Cropley and Zijlstra, 2011) and high levels of neuroticism lead to increased affective rumination, even after controlling for the extend of job stressors (Hamesch et al., 2014). In their review of studies considering affect, Sonnentag et al. (2017) found positive work reflection to be positively related to positive affective states and negative work reflection associated with increased negative affective states, whereas detachment was negatively related to negative affect. In addition to the reduction in negative affect, detachment was also associated with an increase in positive affect in a recent experimental study (Sonnentag and Niessen, 2020). On a within-person level, increased ruminative thinking at night is also associated with higher levels in negative affect the next morning (Wang et al., 2013). On the other hand, mindfulness trainings have been shown to reduce both affective rumination and problem-solving pondering (Querstret et al., 2017). In a study by Hülsheger et al. (2015) detachment remained unaffected by a mindfulness intervention. Other studies however, suggested positive effects from mindfulness interventions on detachment (Michel et al., 2014; Althammer et al., 2021). According to these findings, personality and affective traits are related not only to the extent individuals respond to stressors, but also how: We assume particularly the negative valence inherent in affective rumination leads to stronger associations with individual antecedents of rumination and therefore expect

*H3a*: Neuroticism is positively related to affective rumination and problem-solving pondering, while at the same time negatively associated with detachment.

*H3b*: Positive affect is negatively related to affective rumination and positively related to problem-solving pondering and detachment, whereas,

*H3c*: Negative affect is positively related to affective rumination and problem-solving pondering and negatively related to detachment.

*H4*: Mindfulness is negatively related to affective rumination and problem-solving pondering and positively related to detachment.

Sonnentag et al. (2017) found positive relations of work engagement with morning recovery level and negative relations with morning depletion. Work engagement and psychological detachment jointly predicted end of week affect (Sonnentag et al., 2008). Work engagement predicted subsequent positive work reflection (Sonnentag et al., 2021) and also yielded the strongest links to positive work reflection and problem-solving pondering when compared to other types of work-related cognition (Weigelt et al., 2019a). Similar to work engagement, commitment depicts attitudes toward organization, occupation and form of employment (Meyer et al., 1990). We therefore expect:

*H5a*: Work engagement is negatively related to affective rumination and positively related to problem-solving pondering and detachment.

*H5b*: Commitment is negatively related to affective rumination and positively related to problem-solving pondering and detachment.

Per definition, recovery implies absence of work-related engagement (Geurts and Sonnentag, 2006) and absence of repetitive work-related thought is a condition for psychological detachment (Sonnentag and Fritz, 2015). Full detachment from work entails refraining from work-related worries, problem-solving or planning as well as positive work reflection (Sonnentag and Niessen, 2020) and from positive work-related thoughts (Kinnunen et al., 2017). Since, unlike detachment, both affective rumination and problem-solving pondering are to some extent contaminated with valence and content, we expect that

*H6*: Recovery experiences are negatively related to both affective rumination and problem-solving pondering, whereas detachment is positively related to recovery experiences.

Rumination has been shown to mediate the association between job demands and health (Hamesch et al., 2014). However, less is known about how exactly various job characteristics trigger or buffer different types of rumination. Time pressure was found to be associated with increased negative work rumination (Berset et al., 2011) and impaired detachment (Sonnentag and Fritz, 2015). Interactions with customers provide a potential for negative work-related thoughts after work, especially if these interactions are conflict-ridden (Volmer et al., 2012; Demsky et al., 2019). Job stressors include (potentially) affect-laden conflicts with colleagues or superiors as well as demands related to work content or work organization. However, again particularly the negative valence inherent in affective rumination should yield stronger associations with job stressors compared to problem-solving pondering, which is why we assume

*H7*: Job stressors are positively related to both types of work-related cognition and are negatively related to detachment.

### Outcomes

To continue with potential outcomes of work-related rumination, the following section outlines how the facets of the WRRQ relate to psychological strain, sleep disturbances and somatic symptoms. Lack of recovery is associated with poor well-being (e.g., Sonnentag and Natter, 2004). Positive work reflection is related to increased well-being (Fritz and Sonnentag, 2005), whereas rumination is associated with depressed mood (Morrow and Nolen-Hoeksema, 1990), symptoms of burnout like emotional exhaustion and disengagement (Sousa and Neves, 2021) as well as irritation (Weigelt et al., 2019a). The latter outcome is a psychological strain reaction closely related to the work context (Mohr et al., 2005). Other than positive work reflection, problem-solving pondering does not contain information about the valence of work-related thoughts. However, affective rumination and problem-solving pondering both represent ways in which stressors remain continuously activated *via* cognitive representation. Consistent with the perseverative cognition hypothesis, both should be associated with adverse health outcomes. Given the stronger contamination with valence and content, associations with affective rumination and health outcomes are again expected to be stronger than with problem-solving pondering. We therefore expect

*H8*: Psychological strain indicators are positively related to affective rumination and problem-solving pondering and negatively associated with detachment.

A number of studies noted rumination is an important link between job stressors and both impaired sleep quality as well as problems with falling asleep (Cropley et al., 2006; Zoccola et al., 2009; Berset et al., 2011; Vahle-Hinz et al., 2014; Syrek et al., 2017). Both affective rumination and problem-solving pondering result in cognitive activation, which by definition impairs recovery processes such as sleep. We therefore hypothesize that any form of perseverative cognition impairs sleep onset and sleep quality:

*H9*: Sleep disturbances are positively related to affective rumination and problem-solving pondering and negatively associated with detachment.

Studies found evidence for perseverative cognition to be related to somatic health outcomes, e.g., musculoskeletal, gastrointestinal or allergic problems or complaints related to colds (Verkuil et al., 2010, 2012). We there assume

*H10*: Somatic symptoms are positively related to affective rumination and problem-solving pondering and negatively associated with detachment.

In summary, previous research has examined various antecedents and outcomes of rumination. With the present study, we extend these findings by correlating WRRQ measurements with alternative operationalizations of the investigated antecedents and outcomes (e.g., for neuroticism, mindfulness, commitment, wellbeing, and burnout). On the other hand, we examine the extent to which associations of rumination with antecedents and outcomes known from prior research apply to different facets of rumination operationalized with the WRRQ (e.g., for positive and negative affect, recovery experiences, and job stressors). Effect sizes in the reported studies vary with the outcome of interest. Overall, however, affective rumination has been found to be a stronger predictor of negative health outcomes, whereas associations with problem-solving pondering are less pronounced or absent. In sum, previous research suggests that affective rumination is the most detrimental form of rumination, whereas problem-solving pondering may be less detrimental to recovery (Cropley et al., 2012; Querstret and Cropley, 2012; Hamesch et al., 2014). The relationships proposed here extend prior research, on the one hand by adapting assumptions about relations of rumination with antecedents and outcomes known from related fields of research to the specific work context. On the other hand, they serve to assess criterion, discriminant and convergent validity of WRRQ measurements.

## Study 1: Psychometric properties and measurement invariance of the WRRQ

### Procedure and participants

This multistudy report is a secondary analysis of data previously collected on different research questions considering work-related rumination. Study 1 is based on data from five different samples with a total of *n* = 2,207 respondents, representing a wide range of employees across language, gender, age, and occupation groups. Individual sample characteristics are summarized in Supplementary Table 1: *Sample 1* was drawn from an online split ballot experiment on psychosocial risk assessment (Pauli and Lang, 2022) conducted on Amazon Mechanical Turk (MTurk; Buhrmester et al., 2011). MTurk workers were eligible to participate in the study only if they were currently employed and resided in the United States of America. We used Cloud Research to target a sample of 1,600 employees. After deleting participants with failed attention checks or insufficient effort responding, sample 1 consisted of *n* = 1,509 participants. The data from *sample 2* were collected from *n* = 179 employees who responded to a cross-sectional online survey that was conducted as part of a master’s thesis on work-and health-related outcomes of rumination at a German University (Paetow, 2015). *Sample 3* was taken from the evaluation of an interdisciplinary training on health-related employee management (Schulte et al., 2018). In this longitudinal study, respondents participated in an intervention between measurements, which is why we only use data from the first measurement in the present study (*n* = 238). The *n* = 234 participants from *sample 4* were drawn from a cross-sectional survey on work-related rumination and gender that was conducted as part of a master’s thesis at a German University (Schulz, 2018). *Sample 5* is data from a longitudinal study that was conducted as part of a master’s thesis at another German University (Balzer, 2014). The *n* = 47 employees in this study were asked about their work-related rumination on a daily basis over a period of 1 week. Participants in all studies responded voluntary, anonymously and could terminate participation at any time and without any disadvantage. With the exception of sample 1, which was collected in May 2021, all data were collected before the onset of the COVID-19 pandemic.

### Measures

To assess individual employees’ abilities to mentally unwind from work during leisure time, all samples from study 1 used either the English (sample 1) or German (samples 2–4) version of the Work-Related Rumination Questionnaire (Cropley et al., 2012). This self-report measure is administered on a five-point scale (1 = “rarely or never,” 5 = “very often or always”) differentiating affective rumination from problem-solving pondering and detachment with five items on each subscale. Sample items are “Are you troubled by work-related issues when not at work?” for affective rumination, “I find solutions to work-related problems in my free time.” for problem-solving pondering and “Do you leave work issues behind when you leave work?” for detachment. Note that the original version of the WRRQ (Cropley et al., 2012) used both questions (“Are you troubled by …?”) and statements (“I find solutions to ….”) to operationalize the indicators. In Sample 5, all items were worded as statements. In addition, the samples assed respondents’ gender (except sample 3) and age as well as their job title (except sample 3 and 5). Employees’ scores for affective rumination, problem-solving pondering and detachment are computed by adding the respective subscale items and dividing them by the number of items used in each subscale. Item 1 for detachment was inverse-coded before computing the scale indices. In all of the samples, a number of additional variables related to job demands and employee health outcomes were collected, which we consider for construct validation (see Study 2 below).

### Analytic strategy

One way to test the assumptions from the proposed measurement models is to compare the target model with a number of competing models in order to assess the best model in terms of theoretical plausibility and fit to the data (Fischer and Karl, 2019). For model fit evaluation, we follow best practice recommendations in reporting χ^2^-values with associated degrees of freedom, incremental [comparative fit index (CFI) and Tucker–Lewis Index (TLI)] and residuals based [root mean squared error of approximation (RMSEA), standardized root mean square residual (SRMR)] indices of fit (Jackson et al., 2009). Lai and Green (2016) summarize that RMSEA ≤ 0.05 and CFI and TLI ≥ 0.95 have been deemed as indicator of good fit and RMSEA ≤ 0.10 and CFI and TLI ≥ 0.90 were evaluated as acceptable fit. However, the rule of thumb interpretation of model fit indices are an ongoing discussion (Nye and Drasgow, 2011) and authors have been encouraged to inspect model misspecification rather than disregarding models based on cutoff values (Greiff and Heene, 2017). Accordingly, we evaluate model fit along these proposed values and specify issues with model fit, if necessary.

We use multigroup confirmatory factor analysis (CFA) to test for measurement invariance across groups (Greiff and Scherer, 2018): Configural invariance assumes the number of factors and loading patterns are identical across groups, so that the same sets of items correspond to the same set of factors in all groups. In addition to the assumptions from configural invariance, metric invariance assumes factor loadings are identical across groups. Scalar invariance subsequently assumes factor structure, loadings and item intercepts to be comparable across groups. Given equality constraints are not tenable at any level of invariance testing, restrictions on dedicated items can be removed in order to establish partial invariance, as long as the violation of the theoretically assumed structure is considered to be acceptable (van de Schoot et al., 2012). Partial invariance is thus a reaction to the notion that full scalar invariance is rarely accomplished in applied research (van de Schoot et al., 2015). Since *χ*^2^-differences in measurement invariance testing are sensitive to sample size (Milfont and Fischer, 2010; Cheung and Lau, 2012; Fischer and Karl, 2019), we disregarded *χ*^2^ for invariance testing in favor of comparing changes in relative fit: Simulation studies show Δ CFI is a suitable criterion for determining change in model fit and thus suitable for testing invariance (Chen, 2007), with Δ CFI ≥ −0.01 as an indicator of measurement invariance (Little, 2013).

We report Cronbach’s *α* to assess reliability complemented by McDonalds *ω* as well as Intraclass Correlations Coefficients (ICC) of daily measurements in order to avoid underestimation of reliability due to violations of the strict assumptions of Cronbach’s *α* (Cortina et al., 2020). Factor models were estimated with lavaan version 0.6–8 (Rosseel, 2012) in R version 4.0.2 (RStudio Team, 2021).

## Results

### Factor structure of the WRRQ

Distribution parameters of all 15 items over the respective samples are provided in Supplementary Table 2. Across all samples, item means range from 1.975 to 3.798 (SD between 0.867 and 1.317). According to Mardia’s skewness and kurtosis, items in all samples violated the assumption of multivariate normal distribution (results not shown). However, since the Mardia test is sensitive to even minor deviations from multivariate normal distribution and all item parameters are far from the < 2.0 and < 7.0 thresholds for univariate skewness and kurtosis which are thought to be significant problems for CFA estimates (Curran et al., 1996), we used maximum likelihood estimation with Satorra-Bentler scaled *χ*^2^-test statistic providing robust parameter estimations when distribution assumptions are violated (Finney and DiStefano, 2013). Standardized Factor loadings range from 0.380 to 0.907. Since the root of a standardized factor loading corresponds to the proportion of the variance of the item explained by the factor, explained variance proportions ranged from 61.6 to 95.2%, respectively.

Table 1 shows the comparison of the fit indices across the three different models in the respective samples. In all samples the one-factor solution has the worst fit. When distinguishing work-related cognition from detachment with a two-factor solution in model 2, a slight improvement of the model fit can be observed across all samples. However, the three-factor solution to distinguishing affective rumination from problem-solving pondering and detachment in model 3 showed the best solution with a substantial improvement in model fit indices across all samples. In Sample 1, however, model fit was still unsatisfactory. Modification indices revealed that in this sample items PSP 4 and PSP 5 were correlated contrary to the assumption implied by the model. In the specification of model 4, we therefore included the correlation of the two items, which resulted in a substantial improvement in model fit—notably, without relevant changes to the factor loadings (data not shown).

**Table 1:** Comparison of fit indices from confirmatory factor analyses of different Work-Related Rumination Questionnaire (WRRQ) factor models across studies.

Sample	Model	*N*	*χ* ^2,a^	*df*	RMSEA [90% CI]	SRMR	CFI	TLI
*Sample 1*								
	Model 1	1509	4037.582*	90	0.198 [0.193; 0.204]	0.148	0.670	0.615
	Model 2	1509	3224.037*	89	0.175 [0.170; 0.180]	0.149	0.746	0.700
	Model 3	1509	1468.338*	87	0.115 [0.110; 0.120]	0.100	0.893	0.870
	Model 4	1509	1044.595*	86	0.096 [0.091; 0.102]	0.091	0.925	0.909
*Sample 2*								
	Model 1	179	382.795*	90	0.156 [0.140; 0.172]	0.115	0.709	0.661
	Model 2	179	313.383*	89	0.129 [0.114; 0.145]	0.105	0.802	0.767
	Model 3	179	167.067*	87	0.075 [0.058; 0.092]	0.078	0.934	0.921
*Sample 3*								
	Model 1	238	412.590*	90	0.132 [0.119; 0.145]	0.100	0.753	0.711
	Model 2	238	331.537*	89	0.115 [0.102; 0.129]	0.092	0.812	0.778
	Model 3	238	195.547*	87	0.077 [0.063; 0.092]	0.079	0.917	0.900
*Sample 4*								
	Model 1	234	421.276*	90	0.148 [0.134; 0.163]	0.108	0.747	0.704
	Model 2	234	362.331*	89	0.130 [0.116; 0.144]	0.103	0.808	0.773
	Model 3	234	190.312*	87	0.079 [0.064; 0.095]	0.082	0.930	0.915

Model 1 = one-factor solution, Model 2 = two-factor solution, Model 3 = three-factor solution, Model 4 = Model 3 with Items PSP4 and PSP5 correlated; RMSEA = root mean squared error of approximation; SRMR = standardized root mean square residual; CFI = comparative fit index; TLI = Tucker–Lewis index. ^a^Satorra–Bentler corrected. ^*^p < 0.05.

### Measurement invariance of the WRRQ

We used multigroup CFA to test whether WRRQ measurements are invariant across different groups of employees. Underlying each of the measurement invariance models is the three-factor solution for distinguishing affective rumination, problem-solving pondering, and detachment (model 3). Allowing additional correlations (model 4) between two items in only one group already violates the assumption of equivalent factor structure, i.e., configural invariance. Therefore, we disregard model 4 in favor of model 3 in all invariance tests and recognize that overall model fit may be worse as a result. Table 2 shows the fit indices for the measurement invariance models across languages. To test for invariance across language versions, we merged the data from the German-language surveys (samples 2–4) for comparison with the English version of the WRRQ (sample 1). For the unrestricted models, fit indices for the English sample are worse compared to fit indices for the sum of the German samples. This was expected due to zero-correlations of items in both groups. Accordingly, it is tolerable that fit indices for the configural model marginally exceed rules of thumb for acceptable fit. Recall that the configural invariance model evaluates whether the overall factor structure is equal across groups, i.e., language versions. Introducing additional restricted factor loadings across both groups in the metric invariance model did not lead to a deterioration in CFI ≥ −0.01. Metric invariance was thus accepted. When introducing quality constraints to the intercept across language versions, full scalar invariance was rejected. As recommended by Fischer and Karl (2019), we used modification indices to identify non invariant items and freed the intercepts for items with the strongest effect on model fit (RAR1, PSP1, and DET1), which lead to establishing partial scalar invariance.

**Table 2:** Fit indices for single CFAs and measurement invariance models across languages.

Model	*N*	*χ^2a^*	*df*	RMSEA [90% CI]	SRMR	CFI	TLI	Δ CFI
English	1509	1468.338*	87	0.115 [0.110; 0.120]	0.100	0.893	0.870	
German	651	409.290*	87	0.082 [0.074; 0.090]	0.076	0.918	0.901	
Configural invariance	2160	1911.209*	174	0.106 [0.102; 0.111]	0.088	0.898	0.877	
Metric invariance	2160	2026.292*	186	0.105 [0.101; 0.109]	0.100	0.893	0.879	–0.005
Scalar invariance	2160	2473.905*	198	0.111 [0.107; 0.115]	0.105	0.874	0.866	–0.019
Partial scalar invariance	2160	2208.122*	195	0.106 [0.102; 0.110]	0.102	0.885	0.877	–0.008

RMSEA = root mean squared error of approximation; SRMR = standardized root mean square residual; CFI = comparative fit index; TLI = Tucker–Lewis index. Partial scalar invariance: Intercepts for RAR1, PSP1 and DET1 freely estimated. ^a^Satorra–Bentler corrected. ^*^p < 0.05.

Fit indices for increasingly restrictive models to compare WRRQ measurements across woman and men are shown in Table 3. Results indicate that WRRQ measurement across gender are comparable in terms of general factor structure, factor loadings and item intercepts, leading to the acceptance of scalar invariance across gender.

**Table 3:** Fit indices for single CFAs and measurement invariance models across gender.

Model	*N*	*χ^2a^*	*df*	RMSEA [90% CI]	SRMR	CFI	TLI	Δ CFI
Male	909	730.616*	87	0.099 [0.092; 0.105]	0.093	0.909	0.890	
Female	997	974.196*	87	0.113 [0.107; 0.120]	0.102	0.891	0.868	
Configural invariance	1906	1710.527*	174	0.106 [0.102; 0.111]	0.093	0.899	0.878	
Metric invariance	1906	1751.513*	186	0.103 [0.099; 0.108]	0.094	0.899	0.885	0.000
Scalar invariance	1906	1811.758*	198	0.101 [0.097; 0.105]	0.094	0.897	0.891	–0.002

RMSEA = root mean squared error of approximation; SRMR = standardized root mean square residual; CFI = comparative fit index; TLI = Tucker–Lewis index. ^a^Satorra–Bentler corrected. ^*^p < 0.05.

Based on their age, we assigned employees in the different samples to one of four groups. According to Table 4, model comparisons did not exceed the critical Δ CFI threshold. Thus, scalar invariance was accepted for employees across different age groups.

**Table 4:** Fit indices for single CFAs and measurement invariance models across age groups.

Model	*N*	*χ^2a^*	*df*	RMSEA [90% CI]	SRMR	CFI	TLI	Δ CFI
19 - 34	663	650.839*	87	0.109 [0.101; 0.117]	0.101	0.891	0.868	
35 - 44	623	558.348*	87	0.102 [0.094; 0.110]	0.094	0.911	0.893	
45 - 54	362	295.638*	87	0.093 [0.081; 0.104]	0.087	0.913	0.895	
55 +	251	313.740*	87	0.113 [0.100; 0.127]	0.107	0.901	0.880	
Configural invariance	1899	1803.558*	348	0.104 [0.099; 0.109]	0.091	0.903	0.883	
Metric invariance	1899	1885.408*	384	0.100 [0.095; 0.104]	0.095	0.902	0.893	–0.001
Scalar invariance	1899	2028.585*	420	0.098 [0.093; 0.102]	0.095	0.898	0.898	–0.004

RMSEA = root mean squared error of approximation; SRMR = standardized root mean square residual; CFI = comparative fit index; TLI = Tucker–Lewis index. ^a^Satorra–Bentler corrected. ^*^p < 0.05.

Based on the responses to the open-ended question about their job title, participants were classified into categories of the International Standard Classification of Occupations (ISCO). This was done in a two-step process: First, we used laborR (Kouretsis et al., 2020) to map free-text of occupations to ISCO major groups. In a second step, the result of step one was manually verified and corrected if necessary. In total, 1734 participants could be assigned to an ISCO major group (Hoffmann, 2003). Measurement invariance models across occupations were calculated based on ISCO major groups with substantial group sizes. The results of these analyses based on *n* = 1,589 are presented in Table 5. Introducing constraint factor loadings and intercepts did not lead to considerable deterioration in model fit according to Δ CFI. Thus, scalar invariance was accepted.

**Table 5:** Fit indices for single CFAs and measurement invariance models across occupations.

Model	*N*	*χ^2a^*	*df*	RMSEA [90% CI]	SRMR	CFI	TLI	Δ CFI
ISCO 1	307	288.580*	87	0.100 [0.088; 0.113]	0.087	0.915	0.898	
ISCO 2	736	792.299*	87	0.116 [0.108; 0.123]	0.096	0.871	0.844	
ISCO 3	371	336.261*	87	0.098 [0.087; 0.109]	0.087	0.907	0.888	
ISCO 4	89	151.201*	87	0.095 [0.069; 0.120]	0.097	0.918	0.901	
ISCO 5	86	153.933*	87	0.107 [0.079; 0.135]	0.105	0.886	0.863	
Configural invariance	1589	1725.726*	435	0.107 [0.102; 0.113]	0.087	0.892	0.870	
Metric invariance	1589	1817.926*	483	0.103 [0.098; 0.108]	0.094	0.891	0.881	0.001
Scalar invariance	1589	1957.789*	531	0.100 [0.095; 0.104]	0.095	0.887	0.888	0.004

RMSEA = root mean squared error of approximation; SRMR = standardized root mean square residual; CFI = comparative fit index; TLI = Tucker–Lewis index. ISCO = International Standard Classification of Occupations, ISCO 1 = Legislators, Senior Officials and Managers; ISCO 2 = Professionals; ISCO 3 = Technicians and Associate Professionals; ISCO 4 = Clerks; ISCO 5 = Service Workers and Shop and Market Sales Workers. ^a^Satorra–Bentler corrected. ^*^p < 0.05.

To supplement analyses of invariance across job groups, we compared employees from sample 1 who indicated whether their activities are predominantly intellectual, physical or both. According to Δ CFI, introducing increasing model constraints did not lead to considerable deterioration in model fit. However, model fit was poor in the unconstrained models to begin with (see Supplementary Table 3).

To test for longitudinal invariance, we compared daily WRRQ measurements over the course of 1 week from employees in sample 5. Applying multigroup confirmatory factor analysis to investigate the factor structure of daily measures is in line with prior research on recovery (Bakker et al., 2015). Introducing increasing equality constraints across weekdays did not lead to substantial deterioration in model fit (see Supplementary Table 4). Thus, factor structure as well as loadings and intercepts do not differ across weekdays and scalar invariance was accepted across weekdays. All models in Supplementary Table 4 converged normally. Due to the small sample size, we report additional parameters to assess reliability of WRRQ-measurements in the following section.

### Reliability of the WRRQ

Alpha and omega reliabilities in the respective samples for affective rumination (*α* = 0.82–0.95, *ω* = 0.82–0.95), problem-solving pondering (*α* = 0.82–0.86, *ω* = 0.82–0.86) and detachment (*α* = 0.81–0.84, *ω* = 0.82–0.86) was satisfactory across all samples and seem robust against slight variations of item wording, i.e., whether items are worded as statements (as in sample 5) or operationalized with both questions and statements (see Supplementary Table 5). Intraclass Correlations Coefficients (ICC) of daily measurements from sample 5 indicated affective rumination (ICC1 = 0.45), problem-solving pondering (ICC1 = 0.61) and detachment (ICC1 = 0.61) are relatively stable across the period of one  week. Bland–Altman plots indicate unbiased longitudinal measurements across the period of one working week (see Supplementary Figure 1).

## Study 2: Construct validity of the WRRQ

Study 2 investigated the nomological network of affective rumination, problem-solving pondering and detachment *via* an extensive set of predictors and outcomes of work-related cognition. In addition, correlations with external, discriminant and convergent criteria also serve to assess the validity of WRRQ measurements.

### Procedure and participants

Study 2 is based on data from a total of *n* = 4,002 employees. We combined data on individual employee characteristics, job stressors and employee health outcomes from samples 1 to 4 from study 1 with data from an additional sample 6 of *n* = 1842 employees drawn from a psychosocial risk assessment and occupational health promotion survey at a university in Germany (see Supplementary Table 1).

### Measures

#### Affective rumination, problem-solving pondering, detachment

These three different aspects of work-related cognition were measured using either the English (sample 1) or German (samples 2, 3, 4, and 6) versions of the WRRQ. Sample 6 assessed affective rumination and problem-solving pondering only and did not collect data on detachment.

#### Wellbeing

The WHO-5 Wellbeing Index (*α* = 0.91, *ω* = 0.91) is a unidimensional survey instrument to determine respondents’ well-being over the past 14 days using five items with a five-point frequency scale (“At no time,” “All the time”). An example question is “In the past 2 weeks, I have been happy and in a good mood.” The WHO-5 is a reliable and valid measure (Topp et al., 2015). WHO-5 measurements are language invariant (Sischka et al., 2020) and are suitable to screen for depression (Krieger et al., 2014).

#### Neuroticism

Neurotic personality traits (8 items, *α* = 0.91, *ω* = 0.92) were measured with the dedicated subscale of the Big Five Inventory (John and Srivastava, 1999). Using a five-point rating scale (“Disagree strongly” to “Agree strongly”), respondents were asked to indicate the extent to which they agree with various statements. An example item for the personality dimension neuroticism is: “I see myself as someone who gets nervous easily.”

#### Positive and negative affect

The Positive and Negative Affect Schedule (PANAS; Watson et al., 1988) describes feelings and emotions to which the respondents are asked to indicate to what extent these descriptions generally apply to themselves. Response format is a five-point intensity scale (“very slightly or not at all” to “extremely”). PANAS measures positive affect (*α* = 0.93, *ω* = 0.93) and negative affect (*α* = 0.92, *ω* = 0.92) with 10 items each. Sample items for positive affect are “attentive,” “inspired” or “proud.” Sample items for negative affect are “distressed,” “irritable” or “afraid.”

#### Work engagement

The Utrecht Work Engagement Scale (UWES; Schaufeli and Bakker, 2003) is a 17-item measure for employees’ engagement with their job. The scale maps work engagement on the three subscales vigor (6 items, *α* = 0.86, *ω* = 0.87), dedication (6 items, *α* = 0.89, *ω* = 0.90), and absorption (6 items, *α* = 0.89, *ω* = 0.89). Response format is a 7-point frequency scale ranging from 0 = “Never” to 6 = “Always, every day.” Sample items are “At my work, I feel bursting with energy” for vigor, “I find the work that I do full of meaning and purpose” for dedication and “Time flies when I’m working” for absorption.

#### Mindfulness

The Mindful Attention Awareness Scale (MAAS; Michalak et al., 2008) is a 15-item measure of individuals ability to direct their attention to the present moment and to act with mindfulness (*α* = 0.91, *ω* = 0.91). Response format is a 6-point frequency scale ranging from 1 = “almost always” to 6 = “almost never.” A sample items is “I could be experiencing some emotion and not be conscious of it until some time later.”

#### Depression

The PHQ-2 (Löwe et al., 2002) is a two-item short version of the depression module from the Primary Care Evaluation of Mental Disorders (PRIME-MD) Screening Questionnaire. PHQ-2 utilizes the first two questions on loss of interest and depressed mood over the past 14 days from PHQ-9 to screen for major depression (*α* = 0.88)^1^. Response format is a four-point frequency scale ranging from 0 = “not at all” to 3 = “nearly every day.” The PHQ-2 sum-score can range from 0 to 6 with higher values indicating more severe depressive symptoms. The present study is based on data from a survey with the German translation of the PHQ.

#### Recovery experiences

How individuals unwind and recuperate from work during leisure time was measured using the Recovery Experience Questionnaire (Sonnentag and Fritz, 2007). This self-report measure assesses the four recovery experiences psychological detachment from work (*α* = 0.91, *ω* = 0.91), relaxation (*α* = 0.82, *ω* = 0.82), mastery (*α* = 0.83, *ω* = 0.83) and control (*α* = 0.88, *ω* = 0.88) with four items each on a five-point agreement-scale ranging from1 = “I do not agree at all” to 5 = “I fully agree.” Sample items are “I forget about work” for psychological detachment, “I kick back and relax” for relaxation, “I learn new things” for mastery and “I feel like I can decide for myself what to do” for control.

#### Sleep disturbances

Item 3 of the PHQ-9 addresses issues related to sleep disturbances that can be used as a screener for problems associated with sleep (MacGregor et al., 2012). Based on the wording of the German PHQ-D (Löwe et al., 2002) item 3, “trouble falling asleep” and “trouble staying asleep” over the last 2 weeks were measured with two dedicated items. Response format is a four-point frequency-scale ranging from 0 = “not at all” to 3 = “nearly every day.” The sum of those two items was used to indicate sleep disturbances (*α* = 0.76), with higher values indicating more sleep disturbances.

#### Somatic symptoms

Somatic symptoms were assessed with 11 items from the PHQ (Löwe et al., 2002) scale assessing frequent somatic complaints. The scale assesses whether respondents were bothered by physical symptoms during the past 4 weeks. Response format is a three-point scale ranging from 0 = “not bothered at all,” *via* 1 = “bothered a little” to 2 = “bothered a lot.” Somatic symptoms were calculated as the sum of the items (*α* = 0.73, *ω* = 0.74). The score can range from 0 to 33 with higher values indicating more severe somatic symptoms.

#### Burnout

Burnout was measured using the German version of the Maslach Burnout Inventory (MBI-D; Büssing and Perrar, 1992). The scale consists of 25 items that asses four dimensions of burnout, namely emotional exhaustion (9 items, *α* = 0.85, *ω* = 0.85), personal accomplishment (8 items, *α* = 0.71, *ω* = 0.70), depersonalization (5 items, *α* = 0.64, *ω* = 0.65) and involvement (3 items, *α* = 0.25, *ω* = 0.30).^2^ Response format was a six-point frequency-scale ranging from 1 = “never” to 6 = “often.” Since the present survey was conducted exclusively among supervisors, the original patient-reference of the items was reformulated to refer to employees. Sample items are „ I feel emotionally drained from my work for emotional exhaustion, “I feel I’m positively influencing my employees lives through my work” for personal accomplishment, “I feel I treat some employees as if they were impersonal ‘objects’” for depersonalization and “I feel personally involved with my employees problems” for involvement.

#### Irritation

Cognitive irritation (3 items, *α* = 0.89, *ω* = 0.90) and emotional irritation (5 items, *α* = 0.90, *ω* = 0.91) were measured using the irritation scale (Mohr et al., 2005). Response options ranged from 1 = “Does not apply at all” to 7 = “almost completely true.” A sample item for cognitive irritation is “Even at home I cannot stop thinking about problems from work,” a sample items for emotional irritation is “I react irritably, even if I do not want to.”

#### Job stressors

Job stressors were measured using PsyHealth (Schneider et al., 2019; Kuczynski et al., 2020). This 33-item questionnaire is used in psychosocial risk assessment to assess job characteristics related to work environment (8 items, *α* = 0.78, *ω* = 0.78), social relations with colleagues (4 items, *α* = 0.90, *ω* = 0.90) and with supervisors (4 items, *α* = 0.89, *ω* = 0.90), work intensity (5 items, *α* = 0.80, *ω* = 0.82), task clarity (3 items, *α* = 0.82, *ω* = 0.83), work continuity (3 items, *α* = 0.70, *ω* = 0.71), decision latitude (3 items, *α* = 0.71, *ω* = 0.71) and emotional challenges (3 items, *α* = 0.65, *ω* = 0.69). Response format is a four-point frequency scale ranging from 0 = “at no time or some of the time” to 3 = “most or all of the time.” All job characteristics were coded in a way that higher values indicate more stressful working conditions. An additional measure for work intensity was also included with the subscale of the FIT questionnaire (Fragebogen zum Erleben von Intensität und Tätigkeitsspielraum in der Arbeit, Richter et al., 2000) was developed as a measure to operationalize the two components of the job demands-control model (Karasek, 1979). For the present study, data on work intensity are available which were collected using the six respective items of the FIT (6 items, *α* = 0.83, *ω* = 0.85). Response format was a four-point scale ranging from 1 “no, does not apply” to 4 = “yes, applies.” Higher values on the resulting work intensity sum index indicate more work intensity. A sample item for work intensity is “It is often a lot of work that has to be done by me.”

#### Commitment

The three subscales on organizational commitment from the German “Commitment Organization, Beruf und Beschäftigungsform” questionnaire (COBB; Felfe et al., 2002) were used to assess employees affective commitment (5 items, *α* = 0.86, *ω* = 0.87), continuance commitment (4 items, *α* = 0.73, *ω* = 0.73) and normative commitment (5 items, *α* = 0.75, *ω* = 0.76) to their organization. Response format is a five-point scale ranging from 1 = “does not apply” to 5 = “completely applies” with higher values indicating more commitment. Sample items are “I would be very happy to spend my further working life in this organization” for affective commitment, “At the moment, leaving this organization would be associated with too many disadvantages for me” for continuance commitment and “Many people I care about would not understand or would be disappointed if I left this organization” for normative commitment.

## Results

Means, standard deviations and zero-order correlations of study variables with affective rumination, problem-solving pondering and detachment are presented in Table 6. Affective rumination, problem-solving pondering and detachment were only marginally associated with gender and age. We therefore refrained from reporting partial correlations to control for demographic characteristics. Consistent with the perseverative cognition hypothesis, both types of work-related cognition were related positively with each other (*r* = 0.43, *p* < 0.001), whereas both affective rumination (*r* = −0.57, *p* < 0.001) and problem-solving pondering (*r* = −0.47, *p* < 0.001) were negatively related to detachment.

**Table 6:** Means, standard deviations and zero-order correlations of study variables.

	*M*	SD	1	2	3
*Rumination*					
1. Affective Rumination	2.51	1.01			
2. Problem-solving Pondering	2.86	0.88	0.43***		
3. Detachment	3.39	0.92	–0.57***	–0.47***	
*ANTECEDENTS*					
4. Gender^a^	–	–	0.05*	0.05*	–0.08***
5. Age^b^	40.94	10.88	–0.14***	–0.02	0.05*
*Personality*					
6. Neuroticism^c^	2.50	1.01	0.54***	0.16***	–0.37***
7. Positive Affect^c^	3.30	0.85	–0.33***	0.11***	0.22***
8. Negative Affect^c^	1.54	0.65	0.49***	0.21***	–0.32***
9. Mindfulness^d^	4.27	0.79	–0.50***	–0.17*	0.27***
*Work engagement^e^*					
10. Vigor	2.64	1.10	–0.23**	0.19*	0.2**
11. Dedication	3.18	1.25	–0.16*	0.22**	0.09
12. Absorption	3.03	1.27	–0.11	0.23**	0.06
*Commitment^f^*					
13. Affective Commitment	3.42	0.97	–0.36***	0	–
14. Continuance Commitment	3.21	1.01	0.14***	–0.01	–
15. Normative Commitment	2.45	0.88	–0.03	0.08***	–
*Recovery experiences^g^*					
16. Detachment	3.38	0.83	–0.57***	–0.54***	0.82***
17. Relaxation	3.40	0.69	-0.27***	-0.22***	0.37***
18. Mastery	3.05	0.67	-0.12	0.05	0.09
19. Control	3.79	0.73	-0.22***	-0.18**	0.24***
*Job stressors^h^*					
20. Work environment	0.71	0.55	0.3***	0.08**	–
21. Social relations with colleagues	0.57	0.67	0.41***	0.09***	–
22. Social relations with supervisors	0.86	0.86	0.44***	0.09***	–
23. Work intensity	1.03	0.75	0.49***	0.35***	–
24. Task clarity	0.91	0.76	0.42***	0.17***	–
25. Decision latitude	1.13	0.73	0.29***	-0.04	–
26. Work continuity	1.81	0.71	0.34***	0.25***	–
27. Emotional challenges	0.53	0.59	0.42***	0.18***	–
28. FIT score	15.41	4.18	0.34***	0.37***	–0.31***
*OUTCOMES*					
Psychological strain indicators					
29. Wellbeing^i^	55.78	22.97	–0.52***	–0.13***	0.31***
30. Depression^j^	1.23	1.25	0.51***	0.17***	–0.32***
*Burnout^k^*					
31. Emotional exhaustion	2.35	0.75	0.62***	0.28***	–0.45***
32. Personal accomplishment	4.46	0.50	-0.20**	-0.01	0.18**
33. Depersonalisation	1.97	0.64	0.38***	0.27***	–0.32***
34. Involvement	3.16	0.68	0.10	0.19**	–0.15*
*Irritation^l^*					
35. Cognitive irritation	3.26	1.49	0.70***	0.63***	–0.79***
36. Emotional irritation	2.96	1.36	0.68***	0.32***	–0.51***
*Sleep and somatic outcomes^m^*					
37. Sleep disturbances	1.49	1.46	0.34***	0.08	–0.31***
38. Somatic symptoms	2.91	2.75	0.48***	0.29***	–0.37***

*M* = mean, SD = standard deviation. ^*^p < 0.05, ^**^p < 0.01, ^***^p < 0.001; ^a^*N* = 1906–1907; ^b^*N* = 2,145–2,159; ^c^*N* = 1,508–1,509; ^d^*N* = 168; ^e^*N* = 177; ^f^*N* = 1712; ^g^*N* = 237–255; ^h^*N* = 1,489–1,674; ^i^*N* = 1,509–3,221; ^j^*N* = 408–418; ^k^*N* = 234–253; ^l^*N* = 226; ^m^*N* = 239–254.

### Antecedents of work-related cognition

Neuroticism was positively associated with both types of work-related cognition and negatively associated with detachment. As expected, the high correlation of neuroticism with affective rumination (*r* = 0.54, *p* < 0.001) was stronger compared to the low association with problem-solving pondering (0.16, *p* < 0.001). Positive affect showed a moderate negative relation to affective rumination (*r* = −0.33, *p* < 0.001), but low positive relation to problem-solving pondering (*r* = 0.11, *p* < 0.001) and detachment (*r* = 0.22, *p* < 0.001), whereas negative affect showed low to moderate positive relations with both types of work-related cognition (*r* = 0.21–0.49, *p* < 0.001) but moderate negative relations to detachment (*r* = −0.32, *p* < 0.001). These results support hypotheses H3a and H3b and partially support hypothesis H3c. Mindfulness showed a high negative association with affective rumination (*r* = −0.50, *p* < 0.001), a low negative association with problem-solving pondering (*r* = −0.17, *p* < 0.05) and moderate positive association with detachment (*r* = 0.27, *p* < 0.001), supporting hypothesis H4. As expected, the affective facet of the WRRQ was found to be more strongly associated with personality traits than problem-solving pondering.

Work engagement was negatively related to affective rumination and positively related to problem-solving pondering, supporting hypothesis H5a. However, only vigor showed a low relation to detachment in the way that increased vigor was associated with increased detachment (*r* = 0.20, *p* < 0.01). Affective commitment showed a moderate negative association with affective rumination (*r* = −0,36, *p* < 0.001), whereas continuance commitment showed a small positive associated (*r* = 0.14, *p* < 0.001) and normative commitment was unrelated with affective rumination. Relations between commitment and problem-solving pondering were negligible. No data were collected on the relationship of commitment and detachment. In its general statement, H5b must therefore be rejected, as results indicate relations between rumination and commitment are more complex than hypothesized in H5b.

Except for mastery, all recovery experiences were negatively related to both affective rumination (*r* = −0.57 – –0.22, *p* < 0.001) and problem-solving pondering (*r* = −0.54 – –0.18, *p* < 0.01). Since the presence of work-related thoughts implies absence of psychological detachment and therefore lack of recovery, this relationship can be taken as an indicator of discriminant validity. On the other hand, the high positive relation of detachment measured with WRRQ and recovery experiences (*r* = 0.82, *p* < 0.001) is in line with expectations and an indication of convergent validity. Thus, hypothesis H6 was supported.

In sum, individual employee characteristics showed moderate to strong relations with the affective dimension of rumination (|r| = 0.14–0.57), whereas relations with the problem-solving dimension and detachment were weak to moderate (|r| = 0.08–0.54).

A similar pattern was evident for job stressors. All job stressors showed moderate to strong relations with affective rumination (|r| = 0.30–0.49, *p* < 0.001), whereas only job stressors related to work intensity (*r* = 0.35, *p* < 0.001), task clarity (*r* = 0.17, *p* < 0.001), work continuity (*r* = 0.25, *p* < 0.001) and emotional challenges (*r* = 0.18, *p* > 0.001) showed substantial relations with problem-solving pondering. Data on the relationship of job stressors with detachment were only available from one measurement of work intensity operationalized with the FIT questionnaire (Richter et al., 2000), indicating a moderate negative relationship (*r* = −0.31, *p* < 0.001). In sum, job stressors were generally positively associated with work-related cognition and negatively associated with detachment, while affective rumination generally yielded stronger correlations compared to problem-solving pondering, supporting hypothesis H7.

### Outcomes of work-related cognition

Consistent with expectations, both types of work-related cognition showed moderate to strong relations with psychological strain indicators (|r| = 0.13–0.68), supporting hypothesis H8. Both affective rumination (*r* = −0.52, *p* > 0.001) and problem-solving pondering (*r* = −0.13, *p* > 0.001) were negatively associated with wellbeing, indicating that increased work-related cognition is associated with decreased psychological wellbeing—notably this correlation was high for affective rumination and low for problem-solving pondering. Relations with other strain indicators are positive in the way that increased work-related cognition is related to increased depression, burnout and irritation. The content of irritating thoughts (cognitive vs. emotional) does not make a relevant difference for affective rumination, whereas the relation of problem-solving pondering and cognitive irritation (*r* = 0.63, *p* > 0.001) is high and greater than the moderate relation with emotional irritation (*r* = 0.32, *p* > 0.001). As rumination was described as one facet of irritation (Mohr et al., 2006), the strong positive relation of affective rumination and problem-solving pondering with irritation is an indicator of convergent validity of these subscales, whereas the strong negative relation of detachment and irritation indicates discriminant validity. This is also in line with previous research indicating high overlaps between (lack of) detachment and cognitive irritation (Weigelt et al., 2019a). Accordingly, the high convergence of the two constructs illustrates rumination is as much an indicator of strain as irritation, that has been conceptualized as a job-related strain reaction (Mohr et al., 2006). It should be noted that among burnout indicators, personal accomplishment was unrelated to problem-solving pondering and involvement was unrelated to affective rumination. Only the burnout facet involvement resulted in a low correlation with problem-solving pondering (*r* = 0.19, *p* < 0.01) that is stronger than the low association with affective rumination (*r* = 0.10, n.s.). Apart from this, the findings confirm the negative health effects of work-related cognition, with affective rumination leading to more adverse effects.

Sleep disturbances showed moderate associations with increased affective rumination (*r* = 0.34, *p* > 0.001) and decreased detachment (*r* = −0,31, *p* < 0.001), but—other than expected—were unrelated to problem-solving pondering. Somatic symptoms showed moderate positive relations with affective rumination (*r* = 0.48, *p* < 0.001) as well as problem-solving pondering (*r* = 0.29, *p* < 0.001) and were negatively related to detachment (*r* = −0.37, *p* < 0.001). Thus, hypothesis H9 was partially supported and hypothesis H10 was fully supported.

## General discussion

Recovery research acknowledged work-related cognition as one important answer to the question of what keeps individuals from switching off after work. In this multistudy report, we investigated the psychometric properties, measurement invariance as well as nomological network of the work-related rumination questionnaire (WRRQ), a widely used measure to operationalize affective rumination, problem-solving pondering and detachment as different facets of work-related cognition. Results indicated that the WRRQ is a valid and reliable measure to assess two differential types of work-related cognition as well as detachment. WRRQ measurements are partially scalar invariant across English and German language versions and scalar invariant with respect to gender, age, occupation and over the period of 1 week. Accordingly, measurements from these groups can be considered equivalent, i.e., differences in the expressions of the latent factors are attributable to actual group differences instead of resulting from group-related measurement error. Although prior research compared work-related cognition across groups of workers (e.g., Pravettoni et al., 2007; Pauli and Lang, 2021a) or over time (Syrek et al., 2017; Kinnunen et al., 2019; e.g., Hamesch et al., 2014), to the best of our knowledge, preconditions for such comparisons, i.e., measurement invariance—was never tested. In addition, this study broadened the understanding of how individual employee characteristics, different job stressors and employee health are differentially related to affective rumination, problem-solving pondering, and detachment.

### Psychometric properties of the work-related rumination questionnaire

Study 1 showed that, across different study samples, the three-factor model for differentiating affective rumination, problem-solving pondering, and detachment best fit the data compared to a single-factor and a two-factor model of work-related cognition. The English version of the questionnaire showed deviations from the model implied zero-correlations between items, which lead to worse fit compared to WRRQ measurements in German language. Allowing items “I find thinking about work during my free time helps me to be creative” and “I find solutions to work-related problems in my free time” to correlate unraveled this misspecification and led to improved model fit without challenging the theoretical implications of the measurement model. An explanation for this correlation might be that item wordings in both of these items focus on the way problem-solving pondering is endeavored, whereas other items address anticipation of future work performance or tasks to be done or reflection of past activities. Possibly, respondents to the German version were less receptive to these differentiations. This difference between the English and German WRRQ versions might also explain why only partial scalar invariance was established across languages. Full scalar invariance was accepted when comparing gender, age, and occupation groups. As a consequence, latent variable scores can be compared across these groups. Additional analyses across job activities characterized as intellectually engaging versus physically engaging versus both intellectually and physically engaging did not indicate invariance across groups. One reason might be substantial variation in activities within these groups. Model fit was poor in the unconditional model for the intellectual engagement group to begin with. This could be an indication that the intellectual engagement group may have compromised too wide of a variety of different job activities.

Analyses of longitudinal invariance suggested measurements over the period of 1 week satisfy scalar invariance. Wang and Wang (2020) suggest *n* ≥ 100 per group for multigroup CFA in order to avoid issues with model convergence. In our analyses, all models converged normally. Due to the relatively small sample size, confidence intervals from longitudinal invariance testing suggested reported parameters are associated with quite some degree of uncertainty. Still Cronbach’s Alpha and McDonald’s Omega estimates as well as ICCs support that WRRQ subscales provide reliable measurements of affective rumination, problem-solving pondering and detachment, even across time. WRRQ-measurements are robust against slight variations in items wording, i.e., whether indicators are operationalized as statements or questions. However, for consistency, we recommend sticking with one variant of item wording and provide the German version of the WRRQ in question format in Supplementary Table 6.

Factor loadings were lowest with detachment in all studies. Sonnentag and Fritz (2007) proposed the concept of psychological detachment and showed that recovery experiences can be mapped across the four dimensions detachment, relaxation, mastery and control. Positive correlations with detachment measured with the WRRQ and recovery experiences indicated convergent validity, whereas negative correlations of both types of work-related thought with recovery experiences indicated discriminant validity. Prior research showed that, compared to other recovery experiences, psychological detachment evoked the strongest relations with wellbeing (Sonnentag and Fritz, 2007). This highlights the importance of detachment in the recovery process and is an argument not to disregard detachment when assessing work-related cognition. Thus, the WRRQ might be attractive to researchers interested in contrasting detachment from work-related cognition.

### Nomological network of the work-related rumination questionnaire

Affective rumination and problem-solving pondering are positively associated with each other and are both negatively associated with detachment. This is consistent with theory, since work-related cognition by definition prohibits psychological detachment from work. The modest correlation of affective rumination and problem-solving pondering indicate that both types of work-related thought share common variance. We discuss the implications of this common variance in the future research section. In line with previous findings on the incremental variance explained by each of those two components of work-related cognition (Weigelt et al., 2019a), our analyses indicated differential associations of affective rumination and problem-solving pondering with both antecedents and outcomes.

Personality traits such as neuroticism and positive and negative affect in particular trigger affective components of rumination. This finding is in line with prior research on differential associations of affective rumination and problem-solving pondering with neuroticism (Hamesch et al., 2014; Weigelt et al., 2019a). Previous studies, conceptualized affective states as outcomes of rumination (e.g., Wang et al., 2013; Sonnentag et al., 2017). In addition, our results show that trait affect is an antecedent of rumination as well. Notably, this is primarily true with regard to affective cognition, as indicated by comparably weaker relations of trait affect with problem-solving pondering and detachment.

Regarding job stressors, our results suggested that while affective rumination is related to all job stressors, problem-solving pondering may be triggered mainly *via* job stressors related to work content and work organization. The finding that work intensity in particular is detrimental to detachment is in line with prior research (Sonnentag and Bayer, 2005; Sonnentag and Fritz, 2007). However, the idea that particularly time pressure and overtime work impede recovery *via* prolonged activation, whereas other job stressors might not trigger prolonged activation (Sonnentag and Fritz, 2007) is challenged by these findings: Our analyses show that the affective component of rumination is associated with job stressors *per se*, whereas the problem-solving component is more related to job stressors related to work content and work organization, whereas stressors from work environment or from collegial relationships appear less important. One explanation might lie in the different contamination of the two forms of rumination with valence and content: Because problem-solving pondering is indeterminate in terms of the valence of work-related thoughts, it has been argued that the positive and negative connotations of problem-solving pondering may cancel each other out (Jimenez et al., 2022). Thus, in addition to the content-and task-related demands of work, job stressors might generally carry triggers that evoke employees’ affective responses more than their willingness to engage in problem-solving pondering.

Findings on sleep disturbances are consistent with extensive prior research on positive relations of rumination with sleep onset and sleep quality (Cropley et al., 2006; Zoccola et al., 2009; Berset et al., 2011; Vahle-Hinz et al., 2014; Syrek et al., 2017). Notably, our results indicated the type of work-related cognition altered the relation with sleep disturbances: Other than affective rumination and detachment, problem-solving pondering was unrelated to sleep disturbances. There is evidence of a differential association of affective rumination and problem-solving pondering with fatigue in the way that affective rumination predicted increased fatigue, while problem-solving pondering and detachment predicted decreased fatigue (Querstret and Cropley, 2012). The amount of fatigue induced by differential types of rumination may be one missing link in the relation of affective rumination and problem-solving pondering. However, this finding challenges the assumption that work-related cognition *per se* impedes recovery, whereas relations of affective rumination and problem solving pondering with somatic symptoms supported findings on adverse effects of perseverative cognition (Verkuil et al., 2010, 2012).

In sum, our results support prior research finding affective rumination a stronger predictor of negative health outcomes, whereas outcomes of problem-solving pondering are mixed. Querstret and Cropley (2012) highlighted that when explaining mechanism by which job demands lead to impaired employee health, it is important to identify the type of work-related rumination that leads to ill health rather than rumination *per se*. Furthermore, with reference to the conceptual indifference of problem-solving pondering pointed out by Jimenez et al. (2022), content and valence, in particular should be considered as important predictors of the health relevance of work-related thoughts.

### Limitations and future research suggestions

We investigated the nomological network of affective rumination, problem-solving pondering and detachment *via* an extensive set of antecedents and outcomes of rumination. However, zero-order correlations do not provide in-depth knowledge on causal relations of study variables. Prior research suggested rumination provides a link between job stressors and adverse health outcomes (Brosschot et al., 2005). Future research could examine how rumination alters the relationship between differential job stressors and employee health outcomes, since our results indicate that some job characteristics evoke stronger rumination and might trigger different types of work-related thought. Beyond the scope of the present study, future research should leverage the potential of structural equations models to identify such distinct mechanisms. Except sample 1, all data were collected prior to the COVID-19 pandemic. Although work-related concerns in the context of the pandemic might influence individuals capabilities to switch-off from work, there were no substantial differences in rumination item means across samples collected prior to and during the pandemic. Nevertheless, the pandemic may have influenced other outcome variables collected in sample 1, which is why correlations with WRRQ-factors should be interpreted against this background.

Notably, analyses on longitudinal invariance did not account for fluctuations in job stressors. Sonnentag and Bayer (2005) found that psychological detachment during the evening was negatively associated with day-specific workload. Future research should therefore look at day-to-day covariation of job stressors with work-related rumination. Such analyses are in line with the call for diary methods to investigate employee recovery (Sonnentag and Binnewies, 2013), as recovery may vary across time. Similarly, it can be assumed that rumination varies over time, e.g., assuming that potential stressors cumulate over the course of a working week and decrease over the weekend. Diary studies on relations of unfinished tasks at the end of the week with rumination are one example (Syrek et al., 2017; Weigelt et al., 2019b). Moreover the temporal sequence or interaction of affective rumination and problem-solving pondering is still an open research question: does problem-solving lead to affective rumination when pondering does not lead to a satisfying solution (Querstret and Cropley, 2012; Syrek et al., 2017) or do individuals engage in problem-solving in order to find a solution for their affective ruminative thoughts? Both are potential explanations for the positive cross-sectional correlation of affective rumination and problem-solving pondering.

Finally, work-related cognition was assessed in terms of the extent or valence of work-related thought. In-depth knowledge on the actual content of work-related cognition and its relation to heath related outcomes however is scarce. According to our results, e.g., social relations with colleagues or supervisors are unrelated to problem solving pondering. Syrek et al. (2017) for example found that unresolved tasks which are of particular importance to a person’s self-esteem or professional identity are associated with rumination. Integrating textual responses in online surveys (Rohrer et al., 2017) or web-probing techniques (Behr et al., 2017) might provide valuable insights into what exactly individuals have in mind when they respond to questions on work-related cognition.

## Conclusion

The work-related rumination questionnaire (WRRQ) is a valid and reliable measure to assess three types of work-related cognition: affective rumination, problem-solving pondering and detachment. WRRQ measurements are invariant across gender, age, occupation and time as well as partially invariant across language versions. Affective rumination, problem-solving pondering and detachment differentially relate to antecedents and outcomes and therefore can be used to identify differential mechanism that provide or inhibit recovery from work-related mental efforts.

## Data availability statement

The raw data supporting the conclusions of this article will be made available by the authors, without undue reservation.

## Ethics statement

The study for sample 1 was reviewed and approved by the Ethics Committee at RWTH Aachen Faculty of Medicine. All other samples are secondary analysis of previous studies. Participants in all studies provided their written informed consent to participate in the respective study.

## Author contributions

RP, PG, and JL designed the surveys and collected the data. RP analyzed the data, prepared figures and/or tables and authored the first draft of the manuscript. PG, JL, and MC reviewed and revised drafts of the manuscript. All authors contributed to the article and approved the submitted version.

## Conflict of interest

The authors declare that the research was conducted in the absence of any commercial or financial relationships that could be construed as a potential conflict of interest.

## Publisher’s note

All claims expressed in this article are solely those of the authors and do not necessarily represent those of their affiliated organizations, or those of the publisher, the editors and the reviewers. Any product that may be evaluated in this article, or claim that may be made by its manufacturer, is not guaranteed or endorsed by the publisher.

## Footnotes

^1^*ω* is not reported for two-item scales, since estimation of two factor loadings based on one covariance is not identified.

^2^Differences in the inventory’s subscale-reliabilities are in line with prior research on the MBI’s psychometrics properties, e.g., Golembiewski et al. (1983), Büssing and Perrar (1992), and Neubach and Schmidt (2006).
