# Supplementary material for: Antecedents, outcomes and measurement of work related-cognition in non-work time: A multistudy report using the work-related rumination questionnaire in two languages
Source: Front Psychol. 2023 Mar 2;14:1013744. doi: 10.3389/fpsyg.2023.1013744 (PMC10017545; doi:10.3389/fpsyg.2023.1013744)
Supplement: Supplementary file 1 [file Data_Sheet_1.docx]

# **Supplementary Table 1**

Individual Sub-Sample Characteristics.

| **Sample** | ***N*** | **Design** | **Language** | **Gender** | **Age** | **Occupation** | **Variables** |
| --- | --- | --- | --- | --- | --- | --- | --- |
| 1 | 1509 | experimental | english | 51.1% female | *M* = 40.8 years (*SD* = 11.0) | 6.5% private sector  27.9% public sector | AR, PSP, DET, Wellbeing, Neuroticism, Positive and Negative Affectivity |
| 2 | 179 | cross-sectional | german | 45.8% female | *M* = 37.3 years *(SD* = 10.0) | 37.7% commercial 17.3% technical 11.1% medical/health related 3.9% other | AR, PSP, DET, Work Engagement, Mindfulness, Depression |
| 3 | 238 | cross-sectional | german | n.a. | *M* = 45.1 years (*SD* = 8.80) | leading, shift management or other supervisory positions | AR, PSP, DET, Recovery Experiences, Burnout, Depression, Somatic Symptoms, Sleep Disturbances |
| 4 | 234 | cross-sectional | german | 64.2% female | *M* = 39.0 years (*SD* = 11.0) | n.a. | AR, PSP, DET, Work Intensity, Irritation |
| 5 | 47 | longitudinal | german | 70.2% female | 38.3% 20 - 34 years 61.7% 35 - 59 years | Employees from one pharmaceutical and one auditing company | AR, PSP, DET |
| 6 | 1842 | cross-sectional | german | n.a. | n.a. | University employees: 32.7% administrative departments 67.3% research institutes | AR, PSP, Wellbeing, Job Stressors, Commitment |
| Note. *N* = Sample Size; *M* = Mean; *SD* = Standard Deviation; AR = Affective Rumination, PSP = Problem-Solving Pondering, DET = Detachment. | | | | | | | |

# **Supplementary Table 2**

Means, standard deviations, skewness, kurtosis an factor loadings of individual items in the three factor model across studies.

|  |  | *RAR1* | *RAR2* | *RAR3* | *RAR4* | *RAR5* | *PSP1* | *PSP2* | *PSP3* | *PSP4* | *PSP5* | *DET1* | *DET2* | *DET3* | *DET4* | *DET5* |
| --- | --- | --- | --- | --- | --- | --- | --- | --- | --- | --- | --- | --- | --- | --- | --- | --- |
| *Sample 1 (n = 1509)* | | | | | | | | | | | | | | | | |
|  | Mean | 2.461 | 2.546 | 2.401 | 2.184 | 2.229 | 2.612 | 2.602 | 2.949 | 2.123 | 2.439 | 2.325 | 3.188 | 3.380 | 3.186 | 3.261 |
|  | SD | 1.147 | 1.236 | 1.160 | 1.196 | 1.151 | 1.135 | 1.118 | 1.155 | 1.122 | 1.062 | 1.251 | 1.317 | 1.188 | 1.231 | 1.203 |
|  | Skewness | 0.385 | 0.360 | 0.475 | 0.740 | 0.691 | 0.114 | 0.214 | -0.053 | 0.624 | 0.242 | 0.653 | -0.240 | -0.474 | -0.202 | -0.287 |
|  | Kurtosis | -0.624 | -0.818 | -0.577 | -0.443 | -0.325 | -0.787 | -0.698 | -0.754 | -0.593 | -0.670 | -0.577 | -1.100 | -0.635 | -0.926 | -0.830 |
|  | λ | 0.886 | 0.864 | 0.907 | 0.870 | 0.894 | 0.795 | 0.862 | 0.789 | 0.551 | 0.675 | 0.676 | -0.430 | -0.719 | -0.837 | -0.855 |
| *Sample 2 (n = 179)* | | | | | | | | | | | | | | | | |
|  | Mean | 2.872 | 2.480 | 2.324 | 2.363 | 2.559 | 2.598 | 3.095 | 3.291 | 2.492 | 2.933 | 2.575 | 3.642 | 3.687 | 3.464 | 3.408 |
|  | SD | 0.977 | 1.093 | 1.003 | 1.079 | 1.060 | 1.036 | 0.934 | 0.945 | 1.103 | 0.969 | 1.136 | 1.003 | 0.944 | 1.103 | 1.079 |
|  | Skewness | -0.138 | 0.152 | 0.220 | 0.420 | 0.128 | 0.132 | -0.147 | -0.165 | 0.221 | -0.162 | 0.306 | -0.507 | -0.584 | -0.483 | -0.617 |
|  | Kurtosis | -0.134 | -0.834 | -0.775 | -0.537 | -0.637 | -0.556 | -0.180 | -0.384 | -0.969 | -0.338 | -0.786 | -0.470 | -0.073 | -0.589 | -0.331 |
|  | λ | 0.761 | 0.599 | 0.810 | 0.762 | 0.682 | 0.657 | 0.881 | 0.800 | 0.481 | 0.696 | 0.400 | -0.875 | -0.658 | -0.857 | -0.870 |
| *Sample 3 (n = 238)* | | | | | | | | | | | | | | | | |
|  | Mean | 2.576 | 2.176 | 2.214 | 1.975 | 2.252 | 2.429 | 2.227 | 3.134 | 2.542 | 2.765 | 2.395 | 3.563 | 3.798 | 3.504 | 3.462 |
|  | SD | 0.972 | 1.028 | 0.896 | 0.871 | 0.888 | 1.007 | 0.876 | 0.950 | 1.050 | 0.883 | 1.112 | 1.199 | 0.867 | 1.038 | 1.050 |
|  | Skewness | 0.103 | 0.503 | 0.131 | 0.506 | 0.281 | 0.306 | 0.222 | -0.269 | 0.031 | -0.149 | 0.429 | -0.741 | -0.799 | -0.529 | -0.391 |
|  | Kurtosis | -0.485 | -0.613 | -0.904 | -0.582 | -0.496 | -0.336 | -0.535 | -0.353 | -0.716 | -0.147 | -0.699 | -0.370 | 0.621 | -0.299 | -0.684 |
|  | λ | 0.780 | 0.547 | 0.818 | 0.608 | 0.690 | 0.726 | 0.584 | 0.774 | 0.644 | 0.694 | 0.380 | -0.694 | -0.678 | -0.825 | -0.849 |
| *Sample 4 (n = 234)* | | | | | | | | | | | | | | | | |
|  | Mean | 2.893 | 2.590 | 2.385 | 2.419 | 2.474 | 2.543 | 2.996 | 3.201 | 2.205 | 2.868 | 2.641 | 3.526 | 3.551 | 3.449 | 3.410 |
|  | SD | 0.972 | 1.155 | 0.997 | 1.110 | 1.049 | 1.104 | 1.074 | 1.001 | 1.108 | 0.987 | 1.201 | 1.135 | 1.031 | 1.112 | 1.158 |
|  | Skewness | -0.093 | 0.290 | 0.344 | 0.382 | 0.434 | 0.168 | -0.219 | -0.254 | 0.496 | -0.188 | 0.342 | -0.580 | -0.546 | -0.404 | -0.409 |
|  | Kurtosis | -0.270 | -0.756 | -0.535 | -0.718 | -0.399 | -0.817 | -0.641 | -0.168 | -0.762 | -0.275 | -0.924 | -0.493 | -0.151 | -0.630 | -0.811 |
|  | λ | 0.821 | 0.737 | 0.802 | 0.732 | 0.711 | 0.761 | 0.820 | 0.698 | 0.495 | 0.646 | 0.423 | -0.737 | -0.693 | -0.886 | -0.905 |
| Note. SD = Standard deviation; λ = robust maximum likelihood standardised factor loading estimation; RAR = affective rumination, PSP = problem-solving pondering; DET = detachment. | | | | | | | | | | | | | | | | |

# **Supplementary Table 3**

*Fit indices for single CFAs and measurement invariance models across occupational engagement.*

| *Model* | *N* | *χ²^a^* | *df* | *RMSEA [90% CI]* | *SRMR* | *CFI* | *TLI* | *Δ CFI* |
| --- | --- | --- | --- | --- | --- | --- | --- | --- |
| intellectual | 1042 | 1129521* | 87 | 0.120 [0.114; 0.127] | 0.105 | 0.888 | 0.864 |  |
| physical | 132 | 182048* | 87 | 0.103 [0.082; 0.124] | 0.103 | 0.906 | 0.887 |  |
| both | 332 | 370962* | 87 | 0.109 [0.098; 0.121] | 0.099 | 0.891 | 0.868 |  |
| Configural invariance | 1509 | 1671925* | 261 | 0.116 [0.111; 0.122] | 0.097 | 0.890 | 0.867 |  |
| Metric invariance | 1509 | 1729591* | 285 | 0.111 [0.106; 0.117] | 0.098 | 0.890 | 0.878 | 0.000 |
| Scalar invariance | 1509 | 1827869* | 309 | 0.108 [0.103; 0.113] | 0.100 | 0.887 | 0.885 | 0.003 |
| Note. ^a^Satorra-Bentler corrected; RMSEA = root mean squared error of approximation; SRMR = standardised root mean square residual; CFI = comparative fit index; TLI = Tucker-Lewis index. * p < .05. | | | | | | | | |

# **Supplementary Table 4**

*Fit indices for single CFAs and measurement invariance models across weekdays.*

| *Model* | *N* | *χ²^a^* | *df* | *RMSEA [90% CI]* | *SRMR* | *CFI* | *TLI* | *Δ CFI* |
| --- | --- | --- | --- | --- | --- | --- | --- | --- |
| Monday | 47 | 128.366* | 87 | 0.110 [0.066; 0.148] | 0.108 | 0.899 | 0.878 |  |
| Tuesday | 47 | 125.254* | 87 | 0.109 [0.062; 0.150] | 0.087 | 0.917 | 0.900 |  |
| Wednesday | 47 | 127.630* | 87 | 0.109 [0.065; 0.148] | 0.107 | 0.921 | 0.905 |  |
| Thursday | 47 | 130.217* | 87 | 0.118 [0.073; 0.158] | 0.092 | 0.918 | 0.902 |  |
| Friday | 47 | 130.952* | 87 | 0.115 [0.072; 0.154] | 0.932 | 0.929 | 0.914 |  |
| Configural invariance | 235 | 642.453* | 435 | 0.112 [0.093; 0.130] | 0.087 | 0.918 | 0.901 |  |
| Metric invariance | 235 | 702.858* | 483 | 0.108 [0.090; 0.125] | 0.108 | 0.916 | 0.909 | -0.002 |
| Scalar invariance | 235 | 776.783* | 531 | 0.107 [0.090; 0.122] | 0.111 | 0.910 | 0.911 | -0.006 |
| Note. ^a^Satorra-Bentler corrected; RMSEA = root mean squared error of approximation; SRMR = standardised root mean square residual; CFI = comparative fit index; TLI = Tucker-Lewis index. * p < .05. | | | | | | | | |

# **Supplementary Table 5**

*Cronbach’s Alpha and McDonalds Omega Reliabilities of WRRQ-subscales across samples.*

|  | **α_AR_** | **α_PSP_** | **α_DET_** | **ω_AR_** | **ω_PSP_** | **ω_DET_** |
| --- | --- | --- | --- | --- | --- | --- |
| Sample 1 | 0.95 | 0.86 | 0.82 | 0.95 | 0.86 | 0.83 |
| Sample 2 | 0.84 | 0.83 | 0.84 | 0.84 | 0.82 | 0.86 |
| Sample 3 | 0.82 | 0.82 | 0.81 | 0.82 | 0.82 | 0.82 |
| Sample 4 | 0.87 | 0.82 | 0.84 | 0.87 | 0.82 | 0.86 |
| Sample 5 | 0.85 – 0.93 | 0.80 – 0.90 | 0.90 – 0.96 | 0.85 – 0.93 | 0.81 – 0.90 | 0.91 – 0.96 |

Note. AR = affective rumination, PSP = problem-solving pondering, DET = detachment. For sample 5, the range of reliability parameters is shown based on the respective daily measurements.

# **Supplementary Table 6**

*German Version of the Work-Related Rumination Questionnaire (WRRQ).*

| **Factor** | **Label** | **Item German** |
| --- | --- | --- |
| Affective Rumination | RAR 1 | Werden Sie angespannt, wenn Sie in Ihrer Freizeit über berufliche Dinge nachdenken? |
|  | RAR 2 | Sind Sie verärgert, wenn Sie außerhalb der Arbeit über berufliche Dinge nachdenken? |
|  | RAR 3 | Sind Sie aufgrund von beruflichen Dingen gereizt, wenn Sie nicht bei der Arbeit sind? |
|  | RAR 4 | Ermüdet es Sie, wenn Sie über berufliche Dinge in Ihrer Freizeit nachdenken? |
|  | RAR 5 | Sind Sie aufgrund von beruflichen Dingen beunruhigt, wenn Sie nicht bei der Arbeit sind? |
| Problem-Solving Pondering | PSP 1 | Neigen Sie nach der Arbeit dazu, darüber nachzudenken, wie Sie Ihre Arbeitsleistung verbessern können? |
|  | PSP 2 | Ertappen Sie sich in Ihrer Freizeit dabei, Dinge zu überdenken, die Sie bei der Arbeit getan haben? |
|  | PSP 3 | Denken Sie über Aufgaben nach, die am nächsten Tag bei der Arbeit erledigt werden müssen? |
|  | PSP 4 | Hilft Ihnen das Nachdenken über arbeitsbezogene Probleme während Ihrer Freizeit dabei, kreativ zu sein? |
|  | PSP 5 | Finden Sie in Ihrer Freizeit Lösungen für arbeitsbezogene Probleme? |
| Detachment | DET 1 | Fühlen Sie sich nicht in der Lage von der Arbeit abzuschalten? |
|  | DET 2 | Sind Sie in der Lage, in ihrer Freizeit aufzuhören über berufliche Dinge nachzudenken? |
|  | DET 3 | Fällt es Ihnen leicht, sich nach der Arbeit zu entspannen? |
|  | DET 4 | Schalten Sie von der Arbeit ab, sobald Sie die Arbeit verlassen? |
|  | DET 5 | Lassen Sie berufliche Angelegenheiten hinter sich, wenn Sie die Arbeit verlassen? |

# **Supplementary Figure 1**

*Bland-Altman plots on day-to-day differences in WRRQ-manifest mean subscales.*

**
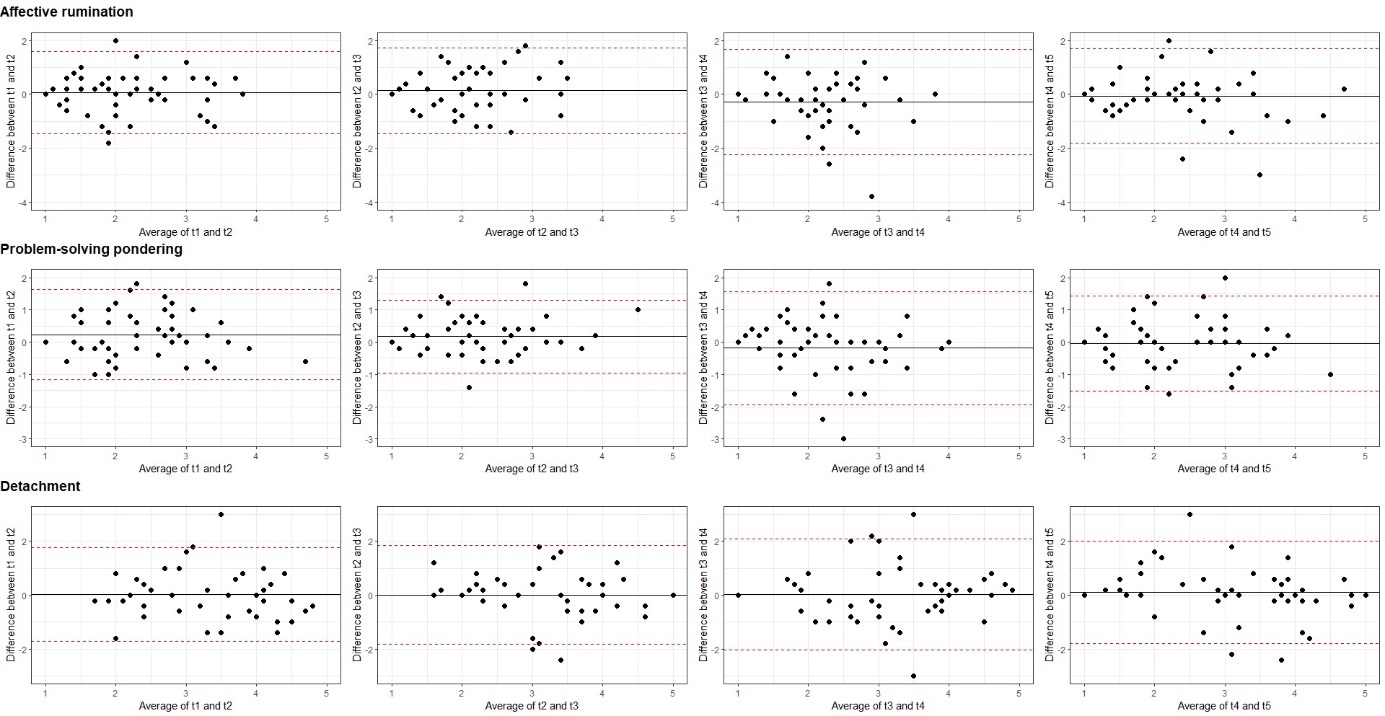
**

Note. Average difference in measurements is indicated by a solid line. 95% of the differences between two consecutive measurements fall within the dashed lines, indicating upper and lower limits of the 95% confidence interval of the average difference in measurements. Average differences in day-to-day measurements on all WRRQ-subscales only marginally deviate from 0 (solid black lines). The scatter around the average differences shows consistent variation across all WRRQ-subscales.
